# Application of Electrospun Nanofiber-Based Electrochemical Sensors in Food Safety

**DOI:** 10.3390/molecules29184412

**Published:** 2024-09-17

**Authors:** Changdong Xu, Jianfeng Tan, Yingru Li

**Affiliations:** College of Intelligent Systems Science and Engineering, Hubei Minzu University, Enshi 445000, China

**Keywords:** electrospun nanofiber, electrochemical sensor, food safety, contaminants, freshness

## Abstract

Food safety significantly impacts public health and social welfare. Recently, issues such as heavy metal ions, drug residues, food additives, and microbial contamination in food have become increasingly prominent. Electrochemical sensing technology, known for its low cost, simplicity, rapid response, high sensitivity, and excellent selectivity, has been crucial in food safety detection. Electrospun nanofibers, with their high specific surface area, superior mechanical properties, and design flexibility, offer new insights and technical platforms for developing electrochemical sensors. This study introduces the fundamental principles, classifications, and detection mechanisms of electrochemical sensors, along with the principles and classifications of electrospinning technology. The applications of electrospun nanofiber-based electrochemical sensors in food safety detection over the past five years are detailed, and the limitations and future research prospects are discussed. Continuous innovation and optimization are expected to make electrospun nanofiber-based electrochemical sensors a key technology in rapid food safety detection, providing valuable references for expanding their application and advancing food safety detection methods.

## 1. Introduction

With the advancement of globalization and the expansion of supply chains, the food industry faces an increasing number of risk factors, including heavy metal residues, pesticide residues, additives, microorganisms, and their toxins [1,2,3,4,5]. These contaminants may originate from agricultural production, processing procedures, or environmental factors. Microorganisms such as *Escherichia coli* and Salmonella can proliferate rapidly in food, leading to foodborne illnesses, while heavy metal and pesticide residues can cause long-term health issues such as neurological damage or cancer. These risks not only threaten consumer health but also have the potential to cause widespread economic and social impacts. Therefore, the development of efficient food safety detection technologies is crucial for ensuring public health [6,7,8,9,10].

**Figure 1:**
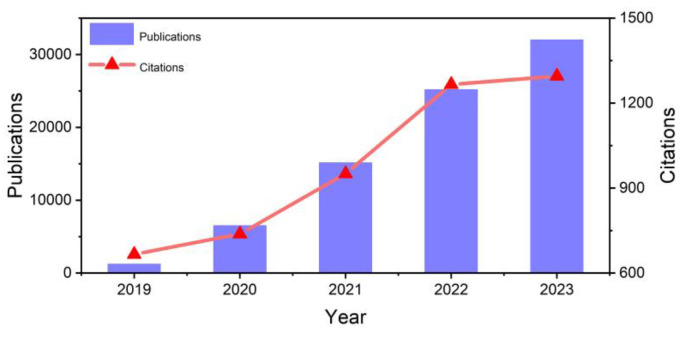
The annual trend of published articles related to the topic “electrochemical sensor food” over the past five years in Scopus.

Traditional food detection methods, such as gas chromatography–mass spectrometry (GC-MS) and high-performance liquid chromatography (HPLC), although providing high precision and reliability, require expensive equipment and complex sample preparation processes. These methods incur high detection costs and involve lengthy analysis procedures, rendering them unsuitable for rapid on-site detection [11,12,13,14,15]. In contrast, electrochemical sensing technology is emerging as a critical technique in the field of rapid food safety detection due to its advantages of speed, low cost, high sensitivity, and portability [11,12,13,14,15]. Figure 1 illustrates the annual trend of published articles related to the topic “electrochemical sensor food” over the past five years in Scopus (2019 to 2023). The results indicate a steady increase in the number of publications related to the application of electrochemical sensors in food analysis. With advancements in nanotechnology and materials science, the performance of electrochemical sensors has been significantly enhanced, allowing for more accurate detection and analysis of harmful substances in food.

Electrospun nanofibers, due to their high surface area and tunable porosity, have demonstrated outstanding application potential in various fields such as filtration, sensor technology, tissue engineering, biomedicine, functional material preparation, and energy storage [16,17,18]. These fibers can be highly customized according to their source materials (including organic, inorganic, carbon-based, and composite materials) and specific structures (such as nonporous, mesoporous, hollow, and core-shell structures) to meet specific application requirements. Particularly in electrochemical sensors, these nanofibers, through specific functionalization treatments, enhance the recognition capability for specific contaminants, thereby improving the sensitivity and selectivity of the sensors [19,20]. Furthermore, the use of nanofibers reduces sensor costs and enhances their portability, enabling rapid on-site detection without the need for complex equipment [21,22,23]. This not only drives innovation in food safety detection technology but also promotes its application globally, contributing significantly to food safety and public health. With continuous technological innovation and interdisciplinary collaboration, a future with enhanced food safety can be anticipated.

## 2. Electrochemical Sensors

**Figure 2:**
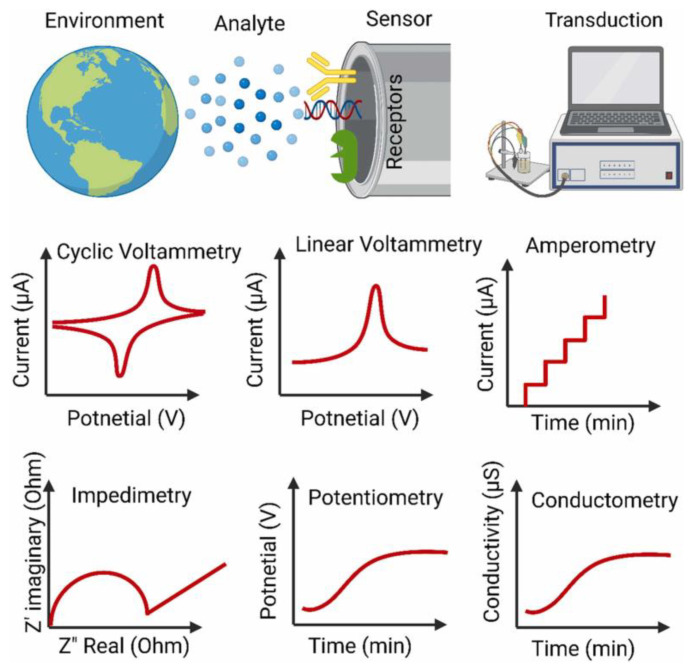
Schematic diagram of electrochemical signal transduction used in electrochemical sensors: voltammetry, amperometry, impedimetry, potentiometry, and conductometry [27].

Electrochemical sensors are extensively researched devices that operate based on interactions between the material on the sensing electrode surface and analytes in the sample, leading to changes in chemical or physical properties [24,25,26]. These changes are converted by a transducer into measurable electrical signals, such as impedance, resistance, potential, or current. By analyzing these signals, the composition and concentration of analytes can be determined, allowing for accurate detection (Figure 2) [27,28]. In essence, the fundamental operating principle of electrochemical sensors is the conversion of chemical signals into electrical signals, relying on redox reactions. Due to their high sensitivity, strong portability, cost-effectiveness, and ease of manufacturing and operation, electrochemical sensors have been widely applied in fields such as food safety, environmental monitoring, and medical diagnostics.

Electrochemical sensors primarily consist of two main components: a recognition element and a signal transduction system (Figure 2) [29,30,31]. The recognition element interacts with the target substance in the sample, causing changes in its physical or chemical properties (e.g., chemical reactions, thermal changes, and photoeffect). These changes are subsequently converted into measurable electrical signals by the signal transduction system. Depending on the target analytes, electrochemical sensors can be categorized into gas sensors, biosensors, and ion sensors. Specifically, gas sensors include semiconductor gas sensors, solid electrolyte gas sensors, electrochemical gas sensors, quartz crystal microbalance (QCM) gas sensors, and catalytic combustion gas sensors. Biosensors encompass DNA sensors, immunosensors, cell sensors, microbial sensors, and enzyme sensors. Ion sensors are classified into optochemical ion sensors, surface acoustic wave ion sensors, field-effect transistor ion sensors, and electrode ion sensors [32,33,34]. The measurement methods of electrochemical sensors include potentiometry, conductometry, voltammetry/amperometry, coulometry, and impedance spectroscopy.

The working electrode is crucial to the performance of electrochemical sensors. While bare electrodes have been the focus of earlier studies, they exhibit limitations such as lower surface area, fewer functional groups, and reduced specificity, hindering the adsorption and catalytic oxidation of target analytes. As a result, research has shifted toward modified electrodes. Surface modifications, achieved through techniques like self-assembly, coating, electrodeposition, and electropolymerization, enhance the electrode’s properties, including hydrophilicity, conductivity, and catalytic activity, thereby accelerating electron transfer and reducing redox overpotential. Electrode modification methods are classified into top-down (e.g., laser etching) and bottom-up approaches (e.g., adding active materials), both aimed at enhancing sensitivity and specificity. Additionally, fabrication methods like screen-printed and carbon paste electrodes are employed. Nanomaterials such as nanoparticles, nanotubes, or nanosheets are used to modify electrode surfaces. It is worth mentioning that the function of metallic nanoparticles in biosensing is closely tied to their physicochemical characteristics and possible interaction with the substances [35,36]. In the field of food safety, these sensors, through improved electrode design, enable rapid and highly sensitive detection of electroactive hazardous substances, providing an effective tool for food safety monitoring.

**Figure 3:**
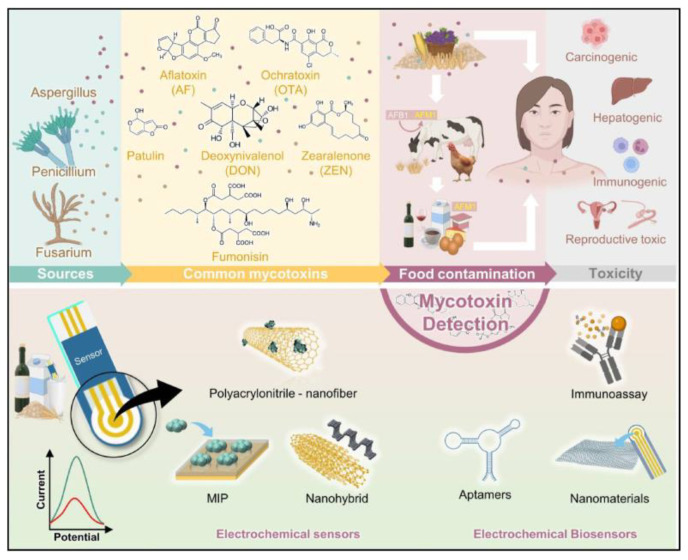
Schematic representation of the development of electrochemical (bio)sensors for mycotoxins detection (MIP: molecularly imprinted polymer) [37].

Electroactive small molecules, such as phenols, alcohols, aldehydes, and compounds containing electroactive groups, as well as metal ions, exhibit excellent electroactivity. Under electrocatalytic conditions, these substances can undergo redox reactions, producing electrical signals through electron transfer. In electrochemical analysis, specific voltages can induce the reduction or oxidation of these electroactive substances, allowing direct detection without traditional affinity recognition methods (Figure 3) [35,36,37]. For components that do not exhibit significant electrochemical activity, their electrochemical responses are weak. To address this, researchers incorporate redox mediators such as ferrocene, potassium ferricyanide, and methylene blue into the detection system, fixing specific recognition elements like enzymes, antibodies, aptamers, or DNA onto the electrode. The specific binding of the target substance to these recognition elements causes changes in the electrical signal produced by the electroactive substances, enabling indirect detection. These affinity-based electrochemical detection methods, such as electrochemical enzyme sensors, electrochemical aptamer sensors, electrochemical immunosensors, and molecularly imprinted sensors, mainly consist of selective biosensitive membranes and electrochemical transducers that convert the biological response into electrical signals [38,39,40]. Loguercio et al. optimized the surface conditions for acetylcholinesterase immobilization, by utilizing a nanocomposite thin film composed of polypyrrole-gold nanoparticles doped with indigo carmine and dodecyl sulfate. The resulting electrochemical enzyme biosensor exhibited a low limit of detection (LOD) and high sensitivity toward the pesticide carbaryl [38]. These sensors demonstrate high sensitivity, high selectivity, low cost, and ease of operation, making them the preferred method for analyzing trace biomolecules and disease markers. With technological advancements, future electrochemical biosensors are expected to further improve in sensitivity, stability, diversity, and practicality, providing stronger technical support for food safety monitoring.

## 3. Electrospinning

**Figure 4:**
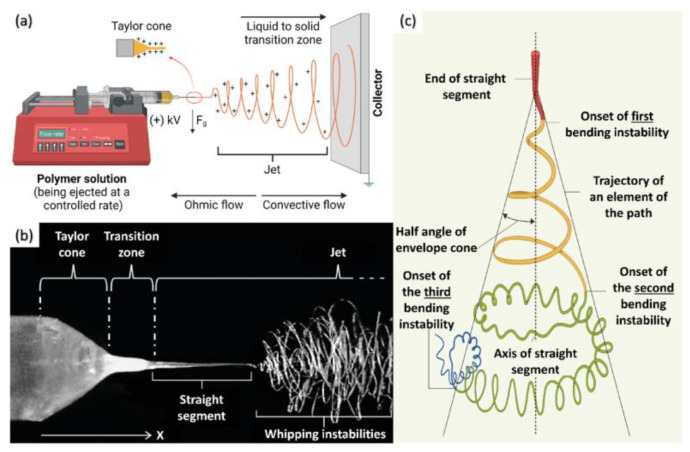
Fundamentals of electrospinning. (**a**) Schematic representation of the electrospinning concept (created with Biorender); (**b**) high-speed photograph outlining the Taylor cone formation, depicting the linear segment of the polymer jet, followed by the whipping jet region; (**c**) the prototypical instantaneous position of the jet path succeeding through the three sequential bending instabilities [41].

Electrospinning is a method for producing one-dimensional nanostructures and is considered one of the simplest and most cost-effective techniques. Originating in the 1930s, it has gained significant attention in both industrial and academic circles since the late 1990s, leading to extensive research and applications [41,42]. Electrospinning is suitable for almost all soluble and fusible polymer materials. To date, over 100 different natural and synthetic organic polymers have been successfully used to produce nanofibers [43,44,45]. The design of electrospinning equipment is simple yet efficient, making it highly valuable for nanofiber fabrication. The equipment primarily consists of three basic components: a high-voltage power supply, a spinneret, and a collector (Figure 4) [41]. During the electrospinning process, the interaction between the liquid surface tension and the applied electric field force is crucial. When the electric field force exceeds the surface tension of the spinning liquid, the charged liquid is ejected from the spinneret as a jet of fibers, which rapidly moves towards the collector. This process can be divided into three main stages: the formation and axial elongation of the fiber jet, the bending instability and thinning of the jet, and the solidification of the jet due to solvent evaporation [46].

The characteristics of the spinning solution, manufacturing parameters, and environmental conditions significantly influence the morphology, diameter distribution, and spinnability of the fibers [47,48]. By precisely controlling these parameters, the electrospinning process can be optimized to produce high-quality fibers with desired properties. Adjustments to relevant parameters, including material properties and manufacturing processes, and the application of various post-treatment techniques, can precisely control the structure and morphology of the resulting nanofiber composites. Specific material parameters involve the characteristics of the polymer solution, such as molecular weight, viscosity, conductivity, polymer chain conformation, surface tension, and solvent type [49]. Process parameters include applied voltage, the distance between the spinneret and the collector, electrode geometry, collector rotation speed, polymer solution feed rate, and environmental factors (such as temperature and humidity) [50]. Post-treatment techniques may include coating, hot pressing, layered surface modification, and plasma treatment [51]. In addition to single-polymer fibers, electrospinning can be used to produce composite nanofibers with various functionalities and properties. Methods for preparing these composite fibers include blended matrix composite nanofibers, core–shell nanofibers, and post-processing modifications [52,53]. By manipulating the material and process parameters, the morphology and properties of composite nanofibers can be precisely controlled. With continuous technological advancements, electrospinning is expected to play an increasingly important role in nanomaterials, filtration materials, and biomedical engineering.

Electrospinning technology can be categorized into various methods based on different manufacturing needs and fiber structure requirements. Each method has distinct characteristics and application scenarios, including wet electrospinning, coaxial electrospinning, multi-needle electrospinning, and needleless electrospinning [54]. Wet electrospinning uses a coagulation bath instead of the traditional solid collector. This method is particularly suitable for handling spinning solutions with low volatility, such as dimethyl sulfoxide or ionic liquids. The coagulation bath not only helps extract non-volatile solvents but also fixes or precipitates the fiber structure, making it a crucial step in the process. However, a major drawback of wet electrospinning is that the produced fibers are usually in the micron range rather than nanoscale, and the complexity of coagulation conditions (such as concentration, type, and temperature) makes the experimental process more complicated [55]. Coaxial electrospinning is similar to traditional electrospinning but uses a spinneret with two concentric needles, each connected to separate spinning solutions controlled by different syringe pumps. The complexity of this technique lies in precisely controlling the relative flow rates of the two liquids to optimize fiber collection conditions. A significant advantage of coaxial electrospinning is the ability to produce fibers with core-shell structures, suitable for applications such as drug delivery, tissue engineering, optics, and smart materials [56,57]. To enhance production efficiency, multi-needle electrospinning technology has been developed. This method increases spinning productivity by using multiple needles simultaneously. Multi-needle electrospinning can produce special composite multilayer fiber materials by loading different spinning coatings or even incompatible polymers. However, as the number of needles increases, jet repulsion and changes in the electric field and charge density at the needle tips can lead to decreased production efficiency and more severe issues. Optimizing this process requires careful consideration of needle configuration, number, and spacing to ensure uniform electric fields and consistent fiber quality [58]. Needleless electrospinning was developed to address the challenges of multi-needle electrospinning and improve production efficiency. In this method, multiple Taylor cones are formed from the spinning solution under high-voltage electric fields and agitation. This technique has proven effective in producing fibers with diameters less than 50 nm and offers high production efficiency. However, the morphology may be lower quality due to reliance on unstable bubbles, and increased solvent evaporation can result in coarser nanofibers.

## 4. Application of Electrospun Nanofiber-Based Electrochemical Sensors in Food Safety

With the acceleration of globalization, food safety and quality monitoring have become crucial issues. Facing challenges such as microbial contamination and chemical and physical pollutants like heavy metals and pesticide residues, electrochemical sensing technology has shown great potential in on-site rapid detection due to its quick response, high sensitivity, and low cost. This technology can accurately detect low concentrations of contaminants in complex food matrices. Electrospun nanofibers, with their small structural dimensions, large surface area, and excellent mechanical properties, are highly advantageous for enhancing the performance of electrochemical sensors [59,60,61]. These nanofibers can be functionalized to recognize specific target molecules, such as proteins, bacteria, or viruses. The high surface area of nanofibers increases the likelihood of contact with target molecules, thereby improving sensor sensitivity. The integration of electrochemical sensors with electrospinning technology not only advances food safety monitoring techniques but also provides an efficient and economical solution for safety monitoring within the global food supply chain, ensuring higher standards of food safety [62,63]. This section will detail the latest research progress of electrospun nanofiber-based electrochemical sensors in food safety, focusing on heavy metal ions, pesticide residues, food additives, microorganisms and their toxins, freshness indicators, and other contaminants. The potential and effectiveness of their application in real-world scenarios will be showcased.

### 4.1. Heavy Metals

**Figure 5:**
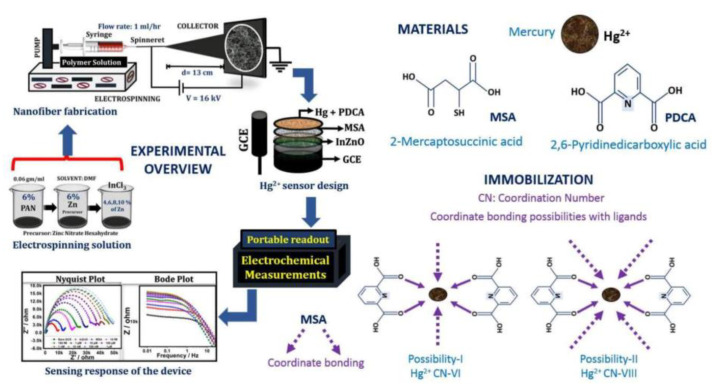
Schematic representation of portable GCE/InZnO electrochemical sensor platform to selectively capture the Hg (II) ions [78].

With the rapid advancement of industrialization, heavy metal pollution has become increasingly prominent. These toxic elements pose serious environmental hazards and can enter agricultural products through the food chain, thereby threatening food safety [64,65]. Heavy metal pollution primarily involves elements with significant biotoxicity, such as lead, cadmium, mercury, and arsenic [66,67,68,69,70,71]. Arsenic is commonly used in pesticides, herbicides, and insecticides, making it easily absorbed by plants and animals [72]. Tang et al. developed a platform by forming polyaniline (PANI) nanosheet arrays on an electrospun Fe-containing carbon nanofiber substrate and self-depositing Au nanoparticles. This platform exhibited a wide linear range (5–400 ppb) and high sensitivity, with a detection limit (LOD) of 0.5 ppb (S/N ≥ 3) [73]. Mercury is one of the most toxic heavy metal pollutants and can easily accumulate in the human body, posing significant health risks [74,75,76,77,78,79,80,81,82,83,84]. Teodoro et al. developed a green electrospun nanofiber composite consisting of reduced graphene oxide, cellulose nanowhiskers (CNW), and polyamide (PA) 6. This nanocomposite demonstrated high sensitivity, stability, selectivity, low LOD of 5.2 nM, and wide dynamic linear range for detecting trace levels of Hg(II) in water [75]. Ehzari et al. created a label-free electrochemical aptasensor based on electrospun polyethersulfone nanofibers and quantum dots designed specifically for detecting Hg^2+^ in juice. Using methylene blue to enhance the specific binding of T-bases to Hg^2+^, this nanocomposite achieved a wide linear response from 0.1 to 150 nM and an LOD of 0.02 nM [76]. Xie et al. prepared carbon nanofibers (CNF) via electrospinning and high-temperature carbonization, followed by hydrothermal loading of platinum nanoparticles. The Pt@CNF nanocomposite was then modified onto a carbon ionic liquid electrode (CILE) and further decorated with gold nanoparticles (AuNPs). A thiol-based aptamer was self-assembled on this structure to create an electrochemical aptasensor (Aptamer/Au/Pt@CNF/CILE). This sensor achieved highly sensitive quantification from 1.0 × 10^−15^ mol/L to 1.0 × 10^−6^ mol/L, with an LOD as low as 3.33 × 10^−16^ mol/L, and was successfully used for real water sample analysis [77]. Rotake et al. designed an electrochemical sensor with indium-doped ZnO nanofibers (InZnO nanofiber) coated with self-assembled monolayers of mercaptosuccinic acid and pyridine dicarboxylic acid. The modified glassy carbon electrode (GCE) sensor exhibited an extremely low LOD of 3.13 fM and high sensitivity, which showed excellent selectivity. This portable electrochemical sensor is the first device capable of detecting Hg(II) at the femtomolar level, making it suitable for on-the-spot detection platforms (Figure 5) [78].

Vilian et al. coated electrospun CNF with petal-like MoS_2_ grown and followed with a facile hydrothermal treatment using thiourea (TA). The proposed MoS_2_-TA-CNF screen-printed carbon electrode (SPCE) showed outstanding electrocatalytic performance and a low LOD of 0.16 nM for Hg^2+^ in acidic media. Its practicality was demonstrated through on-site monitoring of water samples, with recoveries ranging from 86.6% to 110.9%, providing an efficient and economical new approach for monitoring mercury in water [79]. Qi et al. investigated the application of graphene quantum dots-functionalized Ce-ZnO hybrid nanofibers for the electrochemical detection of Hg^2+^. Within the range of 0.1 to 100 μM, LOD of 267 nM [80]. Sadeghi et al. developed a novel electrochemical sensor composed of PA and chromotropic acid (CA) nanofibers mixtures (PANFs-CANFs), exhibiting high electrochemical activity and excellent electrocatalytic performance (α = 0.60, Log Ks = 3.45 s^−1^, Γ = 3.30 × 10^−9^ mmol/cm^2^). The calibration curve was linear in the concentration range of 30 to 450 nM, with an LOD of 9.98 nM and a quantification limit of 29.97 nM. This sensor demonstrated high reproducibility and stability, making it suitable for accurately determining Hg^2+^ levels in drinking water and canned fish samples [81]. Jyothilakshmi et al. optimized the morphology and porosity of titanium dioxide (TiO_2_) nanofibers through electrospinning. They used a GCE modified with silver-coated carbon nanofibers (AgCNF) and nafion as a binder. The TiO_2_/AgCNF/nafion/GCE electrode showed a high sensitivity for Cd^2+^ at 49.98 µA µM^−1^ and an LOD of 12.75 nM [82]. Zhang et al. successfully fabricated flexible Eu-complex/cellulose acetate (CA) nanofibers (Eu/CA nanofibers) luminescent films using electrospinning, exhibited high specificity and sensitivity for Hg^2+^ detection, with an LOD of 3.09 μM and a specificity recognition rate of 97.32%. The nanofiber films could be effectively recycled even after 50 washes [83]. Romaguera-Barcelay et al. employed fluorine-doped tin oxide glass as a substrate, surface-modified with silver nanoparticles (AgNPs) enhanced electrospun CA nanofibers. The sensor demonstrated an LOD of 0.43 μg/L and a sensitivity of 0.33 mA/μg/L for Hg^2+^ [84].

**Figure 6:**
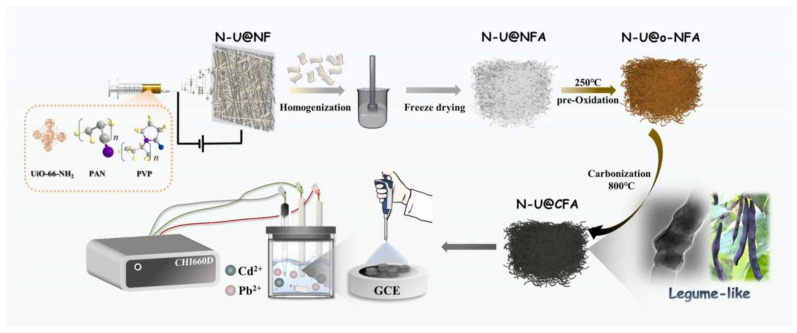
The preparation process schematic diagram for N-U@CFA [87].

The bioaccumulation of cadmium and its transfer through the food chain can compromise food safety and pose significant health risks [85,86,87,88,89,90,91]. Wang et al. developed a novel electrochemical sensor utilizing amino-functionalized metal–organic frameworks (UiO-66-NH_2_) loaded on three-dimensional carbon nanofiber aerogel-modified GCE (N-U@CFA/GCE). This sensor demonstrated extremely low LODs of 0.52 nM and 0.46 nM for Cd^2+^ and Pb^2+^ (Figure 6) [87]. Girija et al. developed nanofibers based on cobalt zinc-zeolite imidazolate frameworks (Co/Zn-ZIF) prepared via electrospinning and applied them for detecting Cd^2+^. This sensor displayed high sensitivity for Cd^2+^ within the range of 100 nM to 1 mM, with a minimum LOD of 27.27 nM. Additionally, the sensor was successfully used to detect Cd^2+^ ions in tap water samples [88]. Ngoensawat et al. successfully fabricated conductive composite fibers containing poly(3,4-ethylenedioxythiophene) (PEDOT) and AgNPs using an innovative emulsion electrospinning technique. These PEDOT/polyvinyl alcohol (PVA)/AgNPs composite fibers were deposited on SPCE, achieving a linear detection range of 10 to 80 ppb with LODs of 6, 3, and 8 ppb for Zn(II), Cd(II), and Pb(II), respectively [89]. Fakude et al. constructed a biotinylated aptamer sensor by immobilizing biotinylated aptamers on acid-activated CNF and further modifying SPCE with streptavidin. This sensor exhibited an LOD for Cd(II) of 0.11 ppb, with a linear response range of 2 to 100 ppb. The aptamer sensor was successfully applied to test real water samples [90]. Yousefi et al. developed a GCE modified with polyindole (PIN)/Mn_2_O_3_ and PIN/Mn_2_O_3_/PANI nanocomposites. It was found that the electrode modified with 5% (PIN)/Mn_2_O_3_/PANI nanofibers exhibited good linearity for Cd^2+^ and Pb^2+^ within the range of 0.05 to 450 μg/L, with LODs of 0.02 and 0.05 μg/L, respectively [91]. Liu et al. combined ZIF-8 with polyacrylonitrile via electrospinning, followed by heat treatment in nitrogen atmosphere. The resulting nitrogen-doped CNF was used to modify electrode surfaces, exhibiting good linear correlation for the detection of Cd(II) and Pb(II) ions, with LODs of 1.11 μg/L and 0.72 μg/L, respectively [68].

Lead and its compounds can cause damage to multiple systems in the body, including the nervous, hematopoietic, renal, cardiovascular, and endocrine systems, leading to anemia, neurological disorders, kidney and liver damage, Alzheimer’s disease, cancer, and more [92,93,94]. Dong et al. constructed an electrochemical sensor based on a flexible cerium metal-organic framework@multi-walled carbon nanotubes/carbon cloth (CeMOF@MWCNTs/CC) for the simultaneous on-site detection of Cd^2+^ and Pb^2+^ in food and water samples. The LODs were 2.2 ppb and 0.64 ppb, respectively. Importantly, this sensing platform was successfully used for the simultaneous detection of Cd^2+^ and Pb^2+^ in grain and water samples, with results consistent with standard methods [92]. Oliveira et al. reported a nanocomposite sensor composed of zinc oxide nanofibers and L-cysteine (ZnO_L-cys), which displayed excellent sensitivity, selectivity, and stability, with a linear range of 10–140 μg·L^−1^ and an LOD of 0.397 μg·L^−1^, achieved recovery rates between 94% and 95% for Pb^2+^ detection in actual samples [93]. Fotia et al. utilized electrospinning technology to combine three different polymers with disodium ethylenediaminetetraacetate (Na_2_-EDTA), a well-known heavy metal chelating agent, to prepare an active layer. This system exhibited a detection range of 10 to 100,000 µg/L for lead, with an extremely low LOD of 0.031 µg/L [94].

### 4.2. Pesticide Residues

**Figure 7:**
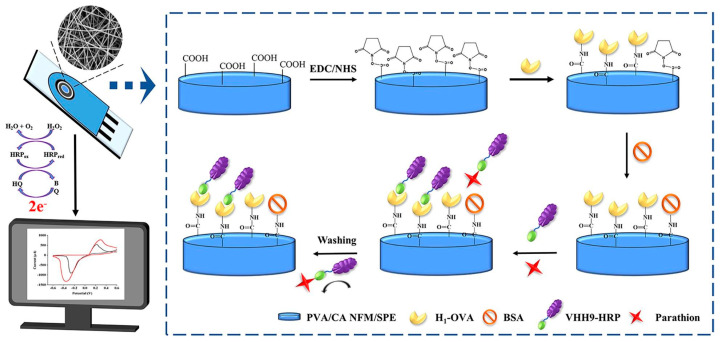
Illustration of stepwise fabrication procedure and sensing mechanism of cross-linked PVA/CA NFM-based immunosensor for parathion detection [103].

Chemical pesticides have significantly increased crop yields, but their overuse has led to contamination of food, the environment, water bodies, and agriculture. Chemical pesticides exhibit cytotoxic, carcinogenic, and mutagenic effects on human health [95,96,97,98,99,100,101,102,103,104,105,106,107,108,109]. Wang et al. developed a novel nanofiber electrochemical sensor, Nafion/polyoxometalate H_3_PMo_12_O_40_ (HPMo)/MoS_2_/Polydiene dimethyl ammonium chloride solution (PDDA)/GCE (Nafion/HPMo/MoS_2_/PDDA/GCE), for the simultaneous detection of clenbuterol and clenbuterol hydrochloride in pork samples. This sensor demonstrated high stability, high sensitivity, and selectivity in a wide range of 1–70 μM for ractopamine (RAC) and 1–350 μM for clenbuterol (CLB) and a low detection limit of 0.056 μM for RAC and 0.085 μM for CLB, respectively [98]. Organophosphorus pesticides are highly toxic, causing contamination and serious health issues through multiple absorption pathways. Shan et al. developed an electrochemiluminescent sensor based on ZIFs electrospun carbon fibers and carbon quantum dots (CQDs). The ZIFs electrospun carbon fibers (ENCF-67) were fabricated using electrospinning and high-temperature calcination. After adsorbing Au nanoparticles, the ENCF-67@Au structure was sequentially modified with CQDs, ENCF-67@Au, and Nafion on the electrode surface, forming the Nafion/ENCF-67@Au/CQDs/GCE sensor. This sensor demonstrated a detection range of 1.0 × 10^−12^ to 1.0 × 10^−7^ M for malathion, with an LOD of 3.3 × 10^−13^ M [99]. Cacciotti et al. employed acetylcholinesterase (AChE) as the biocomponent, immobilized on biopolymer electrospun fiber mats. Poly-hydroxybutyrate-co-hydroxyvalerate (PHBV) was identified as the optimal AChE immobilization carrier, achieving an LOD of 10 ppb and a linear range up to 60 ppb for paraoxon measurement [100]. Migliorini et al. developed a novel electrochemical sensor based on PA6 and polypyrrole (PPy) electrospun nanofibers, surface-modified with chemically reduced graphene oxide (CRGO) and electrochemically reduced graphene oxide. The sensor modified with CRGO exhibited higher conductivity and successfully lowered the LOD for malathion to 0.8 ng/mL [101]. Hu et al. created a label-free electrochemical immunosensor using a functionalized PVA/gelatin-AuNPs nanofiber membrane (PVA/G-AuNPs NFM) for the direct immobilization of nanobodies for quinalphos detection. This sensor showed excellent detection performance with a linear range of 0.06–1000 ng/mL and an LOD of 50.74 pg/mL. In food samples, the quinalphos recovery rates and variation coefficients correlated highly with UPLC-MS/MS methods (R^2^ = 0.9859) [102]. Yin et al. developed an electrochemical immunosensor using crosslinked PVA/citric acid NFM (PVA/CA NFM) and horseradish peroxidase-labeled anti-parathion nanobodies for monitoring parathion pesticide residues in food. The linear response range was 0.01 to 100 ng/mL, with an LOD of 2.26 pg/mL. Real food sample tests showed recovery rates of 96.20% to 114.61%, with a correlation of 0.9964 to UPLC (Figure 7) [103].

Diazinon (DZN) is a widely used organophosphorus pesticide for controlling crop pests and weeds, commonly found in the environment. Topsoy et al. used electrospinning to modify MWCNT-SPE electrodes with poly(ε-caprolactone)/chitosan (CHIT) nanofibers, creating a sensitive tool for detecting low concentrations of DZN residues in food. The electrode exhibited a detection range of 3–100 nM and an LOD of 2.888 nM and successfully detected DZN in real tomato samples [104]. Hatamluyi et al. designed a simple aptamer-based platform for accurate DZN detection. This sensor used nanoscale porous carbon ZIF (NPCZIF) as a support to immobilize the aptamer, which loaded the signal dye methylene blue. The platform was highly sensitive to DZN detection, with an LOD as low as 7.8 fM. Analysis of food and environmental samples showed recovery rates of 95.6–102.6% [105]. Atrazine (1-chloro-3-ethylamino-5-isopropylamino-s-triazine, ATZ) is a widely used triazine herbicide. Drinking water contaminated with ATZ can cause endocrine disruption, and hormonal imbalance, and increase the risk of breast and prostate cancers. P Supraja et al. proposed a sensor using electrospun SnO_2_ nanofibers, achieving ultra-low ATZ LOD of 0.9 zM, with a sensitivity of 4.11 (μA/μM)/cm^2^ and a dynamic detection range from 1 zM to 1 μM. The sensor successfully detected trace atrazine in spiked real water samples, demonstrating its potential for practical applications [106]. Supraja et al. developed an electrochemical sensing platform based on MWCNT-embedded ZnO nanofibers. The sensor achieved high sensitivity and low LODs of 21.61 (KΩ μg^−1^ mL^−1^) cm^−2^ and 5.368 zM, respectively, with a detection range of 10 zM to 1 µM [107].

Chlorpyrifos is a major insecticide in various agricultural products and is associated with endocrine disruption and cancer risk. Dey et al. developed an electrochemical sensor based on Fe-3,5-pyrazoledicarboxylic acid (PyDA)/CNF for the rapid and straightforward determination of organophosphorus insecticides. It exhibited a wide linear detection range (1.0–150 μM), low LOD (15.1 nM), and high sensitivity [108]. Methyl parathion is a widely used agricultural pesticide that causes acetylcholine accumulation, stimulating synaptic receptors and ultimately leading to nervous system damage. Lv et al. prepared Yttrium-stabilized zirconia (YSZ)@rGO nanofibers via electrospinning and thermal reduction, followed by electrodeposition to create PEDOT/YSZ@rGO electrodes for sensitive methyl parathion detection. Under optimal conditions, the LOD was 1.57 ng·mL^−1^, with a linear range of 5 to 4000 ng·mL^−1^ [109]. Wang et al. utilized synthetic MOF nanofibers to fabricate a Burkholderia lipase (BCL)@MOF nanofiber biosensor. The BCL@MOF nanofiber/CHIT/GCE biosensor exhibited high sensitivity for methyl parathion, with a linear range of 0.1–38 µM and an LOD of 0.067 µM. It showed good recovery rates in vegetable sample detection, proving its potential for practical applications [110]. Diphenylamine (DPA) is a significant aniline compound derivative, widely used as a pre- and post-harvest scald inhibitor, fungicide, antioxidant, and pharmaceutical. Kokulnathan et al. reported a TiO_2_/Au nanofibers electrochemical catalyst wrapped with gold heterojunction nanofibers for detecting DPA. The electrode detected DPA within the range of 0.05 to 60 µM, with an LOD of 0.009 µM. Additionally, TiO_2_/Au nanofibers/SPCE were successfully applied in food sample detection [111].

### 4.3. Antibiotics

**Figure 8:**
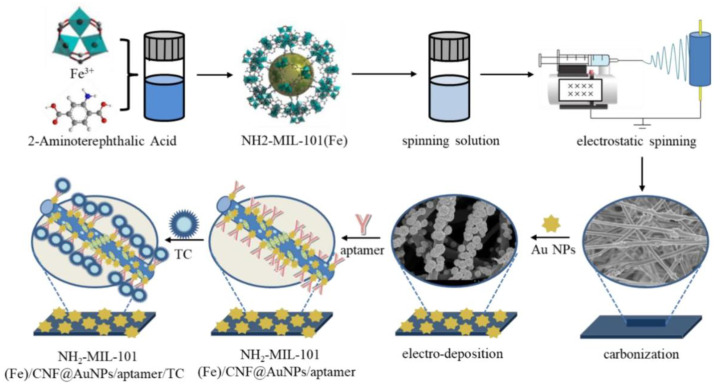
Schematic illustration of the NH_2_-MIL-101(Fe)/CNF aptasensor preparation procedure [115].

Antibiotics are widely used to treat various infectious diseases in humans and animals. The livestock and aquaculture industries worldwide use significant amounts of antibiotics annually, leading to antibiotic residues in food products such as meat, eggs, and fish [112,113]. Huang et al. developed an electrochemical sensor for detecting benomyl based on europium vanadate (EuVO_4_) nanoparticle-modified CNF composites. The EuVO_4_/CNF-modified GCE exhibited a linear range of 0.125 to 23.875–33.81 to 131.375 µM with an LOD as low as 0.00612 µM [114]. Song et al. developed a novel electrochemical aptasensor based on Fe-based MOFs NH_2_-MIL-101(Fe) and AuNPs enhanced CNF (CNF@AuNPs). The sensor showed good linearity in the tetracycline (TET) concentration range of 0.1–10^5^ nM, with an LOD of 0.01 nM (Figure 8) [115]. Mohammadi et al. prepared electrospun polyacrylonitrile (PAN) nanofibers, which were thermally treated to form CNF mats. Gold-modified electrodes were used to immobilize aptamers for TET. This aptamer biosensor exhibited high repeatability, reproducibility, stability, and specificity and was used to detect TET in chicken ham. The detection range was 1 × 10^−9^ M to 1 × 10^−4^ M, with an LOD of 1.2 × 10^−10^ M [116]. Sebastian et al. prepared dual-functional silver-doped zinc ferrite nanoparticles embedded in CHIT-functionalized carbon nanofibers (AgZFO/CHIT-CNF/SPCE) on GCE for the electrochemical detection of TET in environmental and food samples. The sensor showed very high sensitivity, with an LOD of 1 nM and a linear range of 0.2–53.2 μM. The feasibility was evaluated in real samples, such as chicken feed, shrimp, milk, soil, and wastewater, with high recovery rates [117].

Nitrofurazone is widely used for preventing and controlling bacterial infections. However, long-term or high-dose use leads to residues that can cause serious health problems. Kokulnathan et al. developed an electrochemical sensor based on TiO_2_/Au nanofibers (NFs) with O-C_3_N_4_ nanosheets (NSs), with a wide working range (0.008–105 μM), low LOD (0.001 μM), and high sensitivity (1.40 µA µM^−1^ cm^−2^). The sensor performed excellently in real sample detection [118]. Wei et al. synthesized Bi_2_O_3_ nanomaterials combined with Au nanoparticles using electrospinning to create a high-efficiency, sensitive electrochemical sensor for ampicillin. The sensor had a detection range of 1 nM to 10 mM, an LOD of 0.88 nM, and an R^2^ of 0.9914, showing good selectivity, reproducibility, and stability. The sensor was successfully used to determine ampicillin in tap water and milk [119]. Mariappan et al. synthesized Fe_2_WO_6_ via hydrothermal methods and prepared CNF using electrospinning. The CNF/Fe_2_WO_6_ composite was used to modify GCE for the electrochemical detection of metronidazole, which showed an LOD for Metronidazole of 0.013 μM, a sensitivity of 1.55 μA µM^−1^ cm^−2^, and a linear range of 0.01–1792 μM. The electrode also exhibited excellent performance in real sample analysis [120]. Vilian et al. developed a novel material, CNF–NiCo-LDH, consisting of conductive nickel–cobalt layered double hydroxide (NiCo-LDH) grown on electrospun carbon nanofibers (CNF). The CNF–NiCo-LDH, deposited onto a GCE, exhibited a wide linear response for metronidazole reduction, with concentrations ranging from 3 to 57 nM, an extreme LOD of 0.13 nM, and a high sensitivity of 1.294 μA nM^−1^ cm^−2^ [121]. A nanocomposite material based on dysprosium vanadate (Dy(VO_4_)) was designed by Muthukutty et al. for the determination of the antiprotozoal drug metronidazole at low potentials. The sensor shows a wide dynamic linear range (1.5–1036.9 µM), a low detection limit (6 nM), and a limit of quantification (LOQ) of 0.022 µM, along with high sensitivity (1.12 μA μM^−1^ cm^2^). [122]. Shoba et al. reported the preparation and characterization of novel conductive materials PANI-CoPc-fur and PANI-CoPc-fur-f-MWCNTs (CoPc-fur=tetra-4-(furan-2-methylthiophthalocyaninato)Co(II), f-MWCNTs=carboxylic acid-functionalized multiwalled carbon nanotubes), which were fabricated into electrospun nanofiber composites (ENFs-2) adsorbed on GCE and covered with a Nafion layer (GCE|ENFs-2 nanofibers). The electrode exhibited a detection range of 10 to 200 μM, with an LOD of 0.094 μM and a quantification limit of 0.28 μM. Its analytical performance was comparable to liquid chromatography–mass spectrometry [123].

### 4.4. Food Additives

Food additives (antioxidants, bleaching agents, sweeteners, preservatives, colorants, and thickeners) can extend the shelf life of food and enhance its color, aroma, taste, and nutritional value; however, excessive use can pose significant health risks [124,125,126]. Excessive intake of nitrites can lead to diseases such as cancer and hypertension. Wang et al. synthesized one-dimensional honeycomb-like carbon nanofibers (HCNF) using electrospinning technology. By modifying SPCE with Bi/HCNF, the constructed Bi/HCNF electrode (Bi/HCNF-SPCE) exhibited high sensitivity (1269.9 μA mM^−1^ cm^−2^) and a low LOD (19 nM) in the range of 0.1 to 800 μM. This sensor has been successfully applied to detect nitrites in food and environmental samples [127]. Le et al. successfully fabricated a composite of worm-like gold nanowires (Au WNWs) assembled on a high-quality carbon nanofibers-graphene (CNFs–Gr) hybrid network. The nitrite detection demonstrated a wide linear detection range (1.98 µM–3.77 mM), high sensitivity (836 μA cm^−2^ mM^−1^), and low LOD (1.24 µM) [128]. Ranjith et al. synthesized FeWO_4_ bimetallic nanofibers through electrospinning and surface-decorated them on Ti_3_C_2_T_x_ MXene, forming an MXene-FeWO_4_ ternary structure. This material exhibited an LOD of 0.42 nM, a linear range of 4–147 nM, and a sensitivity of 0.3799 μA nM^−1^ cm^−2^. The MXene-FeWO_4_-GCE nanocomposite showed excellent recovery and selectivity when detecting rutin in human serum, orange juice, and tea samples [129]. Chen et al. synthesized single-walled carbon nanohorns/carbon nanofiber (SWCNHs/CNF) composites using electrospinning technology and modified a GCE to develop an electrochemical sensor. This sensor displayed a good response to luteolin in the concentration range of 0.01 to 50 μM, with an LOD of 3.714 nM [130]. Vanillin (VNL) is a flavoring agent, and excessive VNL can cause liver and kidney damage. Vinoth et al. developed a rapid electrochemical sensor based on rhombohedral LaCoO_3_ (LCO) and LCO@CNF hybrid composites. The sensor exhibited a wide linear concentration range of 0.01–1670 μM, a low LOD of 4.67 nM, and a sensitivity of 9.859 μA μM^−1^ cm^−2^. This system was successfully applied for the rapid detection of VNL in real food samples such as vanilla ice cream and cocoa biscuits [131]. Nixon et al. designed an electrochemical sensor based on La_2_NiO_4_-functionalized carbon nanofibers (La_2_NiO_4_-CNF). The sensor exhibited excellent performance for the detection of VNL, including a low LOD (6 nM), and a wide linear range (5 nM–3035 µM). The modified electrode material’s practical application was verified by testing chocolate and ice cream samples [132].

**Figure 9:**
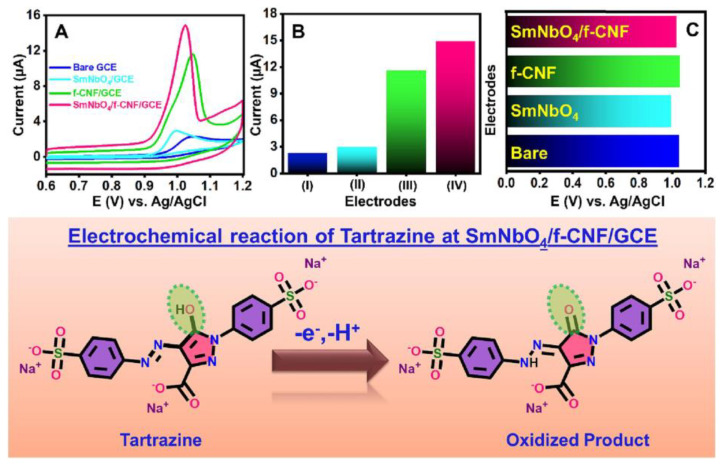
(**A**) CV curves obtained a bare and different modification of GCE; (**B**) histogram of different electrodes with corresponding peak current values; (**C**) histogram of different electrodes with corresponding peak potential values; all the above experiments were performed in 0.1 M phosphate buffer as an electrolyte; (**Bottom**) electrochemical oxidation of TRZ at SmNbO_4_/f-CNF/GCE [135].

Tartrazine (TRZ) and sunset yellow (SY) are widely used food colorants, but they pose potential health risks, including respiratory problems, migraines, and skin allergies. Gokulkumar et al. developed a novel sensor for TRZ detection based on a nickel phosphide composite material modified with functionalized CNF (Ni_2_P@f-CNF). The sensor demonstrated a low LOD (0.011 µM), and a wide linear range (0.01 to 1875 µM). In practical applications, the sensor successfully detected TRZ in soft drinks and liquid soap, with recoveries ranging from 97.45–99.37% and 98.26–99.01%, respectively [133]. Jenisha Daisy Priscillal et al. developed an innovative TRZ electrochemical sensing platform by decorating CNF with nanoscale SmNbO_4_ electrode modifiers. The sensor exhibited a low LOD (2 nmol/L), a wide linear range, good selectivity, and functional stability (Figure 9) [134]. Radha et al. developed an electrochemical sensor using neodymium vanadate (NdVO_4_) nanoparticles embedded in functionalized carbon nanofiber (F-CNF)-modified electrodes. The NdVO_4_@F-CNF sensor’s LOD was 0.0011 μM, with a linear response to TRZ in the range of 0.05–271.6 μM. The sensor was successfully used to detect TRZ in orange juice and jelly samples [135]. Ansari et al. developed a composite film based on ruthenium oxide nanofibers and polysulfonic acid (RuO_2_ nanofibers/PSSA) for the precise detection of SY in food. RuO_2_ nanofibers were first prepared by electrospinning, followed by the electrochemical deposition of RuO_2_ nanofibers/PSSA composite on a commercial carbon paste electrode. The sensor exhibited a linear relationship in the ranges of 0.0005–9.0 μM, with an LOD of 0.38 nM [136].

Monosodium glutamate (MSG) is a commonly used food additive, and excessive consumption can lead to health issues such as headaches. Atilgan et al. developed a biobased MSG detection system using dendritic polymer-modified montmorillonite (Mt) with poly-ε-caprolactone (PCL) and CHIT-based nanofibers, combined with glutamate oxidase (GluOx). The linear range of the PCL–CHIT/Mt/GluOx system for MSG determination was 0.025 to 0.25 mM, with an LOD of 7.019 µM [137].

### 4.5. Microorganisms and Toxins

Foodborne diseases caused by bacterial contamination have become a significant threat to human health. Approximately 31 pathogens have been confirmed to cause foodborne illnesses, including Salmonella, Norovirus, Listeria, Staphylococcus aureus, and *Escherichia coli* [138,139,140]. Wang et al. modified bare SPCE through a two-step drop-casting method, initially depositing polyacrylonitrile-derived electrospun CNF and subsequently immobilizing *E. coli* bacteriophages (bacteriophages/CNF). This biosensor achieved a response within 10 min and exhibited a linear response in the *E. coli* concentration range of 10^2^–10^6^ CFU/mL, with an LOD of 36 CFU/mL [141]. Shaibani et al. reported a simple, rapid, and cost-effective *E. coli* detection technique utilizing a light-addressable potentiometric sensor integrated with electrospun PAA/PVA hydrogel nanofibers as the sensing layer (NF-LAPS). The exceptional Nernstian response of 74 mV/pH change in NF-LAPS provided high sensitivity to *E. coli*, with a theoretical LOD of 20 CFU/mL. The addition of D-mannose ensured specificity in detecting *E. coli* in drinking water applications [142]. Soares et al. developed a nanostructured electronic tongue for detecting S. aureus in milk. The electronic tongue comprised flexible electrodes coated with curcumin carbon dots and zein electrospun nanofibers. The zein sensor unit demonstrated high sensitivity with an LOD of 0.83 CFU/mL. Through interactive document mapping (IDMAP) and machine learning, the electronic tongue could distinguish between milk from mastitis-infected cows, healthy cows, and milk spiked with interferents, achieving an 80.1% accuracy in diagnosing mastitis using a decision tree algorithm [143]. Zhang et al. proposed an electrochemical sensor based on electrospun CA nanofiber-modified paper-based screen-printed (PBSP) electrodes. The CA nanofibers were directly collected on the PBSP electrodes via electrospinning. The detection ranges for glucose, Ag85B protein and *E. coli* O157 were 1 nmol/mL–100 μmol/mL, 100 fg/mL–10 μg/mL, and 1.5 × 10^2^–10^6^ CFU/mL, with LODs of 0.71 nmol/mL, 89.1 fg/mL, and 30 CFU/mL, respectively [144].

Mycotoxins are significant secondary metabolites produced by fungi and microorganisms, posing serious teratogenic, carcinogenic, and nephrotoxic risks to animal and human health. Zearalenone (ZEN), a non-steroidal dietary estrogen produced by Fusarium species, is commonly found in maize, barley, and other cereal products. Moradi et al. developed a nanofiber-modified pencil graphite electrode via electrospinning technology, demonstrating a linear response to ZEN concentrations ranging from 5 to 100 nm [145]. Huang et al. developed an electrochemical biosensor based on Bi_2_S_3_ nanorods and CNF. The sensor exhibited excellent electrochemical properties, including a wide linear detection range and low LOD, showing significant potential for ZEN detection in real agricultural environments [146]. Rahmani et al. developed an electrochemical aptasensor for aflatoxin M1 (AFM1) detection in milk, based on electrospun CNF mats modified with electrodeposited AuNPs and thiol-modified single-stranded DNA (ss-HSDNA). The ss-HSDNA/AuNPs/ECNF demonstrated an LOD as low as 0.6 pg/mL, a wide linear range of 1–100 pg/mL, high sensitivity, and excellent stability and reproducibility [147]. Ebrahimi Vafaye et al. utilized penicillin aptamers as specific recognition elements on a CNF mat with AuNPs, achieving high selectivity, good stability, and excellent reproducibility with a linear range of 1–400 ng/mL and an LOD of 0.6 ng/mL [148].

**Figure 10:**
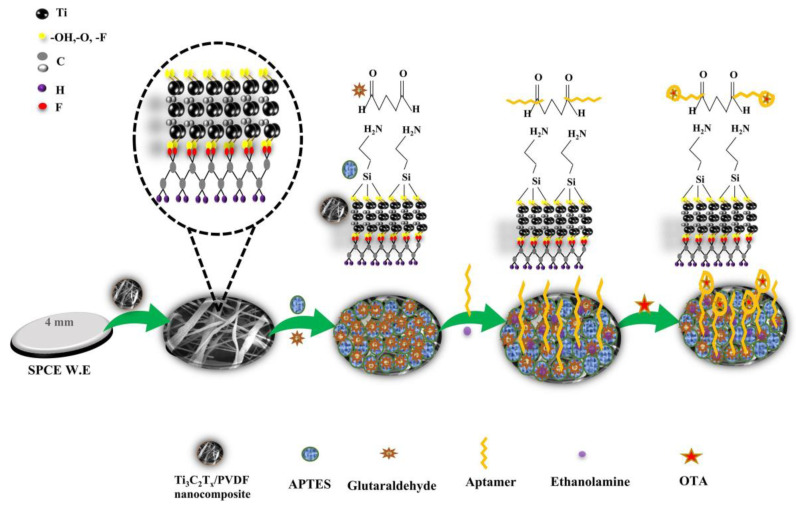
Schematic illustrations of the aptasensor fabrication and working mechanism [150].

Ochratoxin A (OTA) is harmful to human and animal health, necessitating sensitive and selective detection methods. El-Moghazy et al. developed an innovative ultrasensitive electrochemical aptamer sensor for detecting OTA in cold brew coffee by combining nanofibers, electrochemical methods, and aptamer technology. The aptamer sensor used activated silanized cellulose NFM as the support matrix for methylene blue redox probe-labeled aptamers. The sensor exhibited high sensitivity and specificity for OTA in the range of 0.002–2 ng mL^−1^, with an LOD of 0.81 pg mL^−1^. The sensor successfully detected OTA in cold-brew coffee samples without any pretreatment [149]. Al-Dhahebi et al. developed an ultrasensitive electrochemical aptasensor electrode using electrospun MXene/polyvinylidene fluoride (Ti_3_C_2_T_x_/PVDF) nanofiber composites. The composite material was coated on SCE and chemically functionalized with salts and aldehydes to load aptamers. The optimized aptasensor exhibited high sensitivity to OTA within a dynamic concentration range of 1 fg/mL to 1 ng/mL, with an LOD of 2.15 fg/mL and a quantitation limit of 6.52 fg/mL, and high selectivity. The aptasensor successfully detected OTA in grape juice samples at femtogram per milliliter levels (Figure 10) [150]. Dos Santos et al. fabricated a nanostructured immunosensor using a low-cost cut-printing method, modified with zein/PPy electrospun nanofibers. The nanofibers were deposited on the working electrode during the electrospinning process, and anti-aflatoxin B1 (AFB1) monoclonal antibodies were covalently immobilized on the surface. The immunosensor exhibited high sensitivity to AFB1, with a linear detection range of 0.25 to 10 ng/mL and a theoretical LOD of 0.092 ng/mL [151].

### 4.6. Food Freshness

Food spoilage is primarily caused by microbial activity, which produces total volatile basic nitrogen, including ammonia, dimethylamine (DMA), and trimethylamine (TMA) generated by amino acid decomposition. Thus, detecting ammonia content in packaged food samples can effectively indicate food spoilage by measuring bacterial activity [152,153,154,155]. Gas sensors, capable of detecting specific gases or continuously measuring gas composition and concentration in designated areas, effectively monitor food quality and freshness. Li et al. prepared C-N/SnO_2_-based hierarchical microspheres with tunable morphology via a simple electrospinning and subsequent calcination process. The constructed C-N/SnO_2_/ZnO/Au composite material demonstrated a maximum response of approximately 1970 to 50 ppm triethylamine (TEA) molecules. Importantly, the novel C-N/SnO_2_/ZnO/Au sensor effectively detected low concentrations of volatile substances released during fish storage at 80 °C [156]. Rianjanu et al. reported the fabrication and functionalization of electrospun CA-doped polyvinyl acetate (PVAc/CA) nanofibers on a QCM chip. The PVAc/CA nanofiber sensor exhibited a sensitivity of 85.4 Hz ppm^−1^ and an LOD of 19 ppb to TMA vapor [157]. Ma et al. synthesized one-dimensional MoO_3_ nanofibers and two-dimensional Ti_3_C_2_Tx MXene nanosheets via electrospinning and chemical etching methods. The Ti_3_C_2_T_x_ MXene-MoO_3_ composite exhibited excellent room temperature response characteristics to TMA gas, with high sensitivity (response value of up to 4 to 2 ppm TMA) and fast response and recovery times (10 s/7 s) [158]. Qu et al. designed and constructed PAN/PANI fiber membranes with abundant active sites using electrospinning technology. The sensor fabricated from PAN/PANI fiber membranes (with a PAN/PANI mass ratio of 1:0.42) exhibited ultra-high sensitivity (LOD below 6 ppb) and excellent repeatability (RSD = 4.7%, n = 10) to TMA [159]. Li et al. developed a flexible TMA sensor based on In_2_O_3_ nanofibers using electrospinning and deposition techniques. The sensor detected TMA gas as low as 1 ppm at 80 °C, with a response of 3.8. Furthermore, the sensor demonstrated rapid response (6 s) and recovery (10 s) performance [160]. Yang et al. prepared a series of Sn-doped NiO hollow nanofibers through simple electrospinning and thermal treatment. The sensor achieved the highest response value of approximately 16.6 to 100 ppm TEA [161]. Cai et al. used polyoxometalates (POM) as auxiliaries to prepare ZnWO_4_/ZnO heterostructured nanofibers via a simple combination of electrospinning and thermal oxidation. For 50 ppm TEA, the relative response reached 108.5, with an LOD as low as 150 ppb [162]. Ma et al. prepared tunable In_2_O_3_ hierarchical one-dimensional electrospun fibers and in situ grown octahedral-like particles via single-nozzle electrospinning. The hierarchical In_2_O_3_ sensor showed good gas sensitivity to 50 ppm TEA at 25 °C, with a response value of approximately 11.2, reaching 87.8 at the optimal working temperature of 40 °C [163]. Xie et al. fabricated one-dimensional (In_2_O_3_) nanofiber/two-dimensional (Ti_3_C_2_T_x_) MXene composites via electrospinning and hydrothermal methods. At 120 °C, the sensor exhibited a response value of 12.44 to 50 ppm TMA. The sensor also demonstrated rapid response/recovery times of 4/3 s [164]. Wang et al. prepared SnO_2_ nanofibers modified by Pd@ Co-MOF (ZIF-67)-derived trace PdO and Co_3_O_4_ nanoparticles by electrospinning and calcination. The sensor based on 0.012 wt% PdO-Co_3_O_4_-SnO_2_ exhibited a response value of 14 to 20 ppm TEA at 240 °C, with a response time of 3 s and a minimum detection concentration of 1 ppm [165].

**Figure 11:**
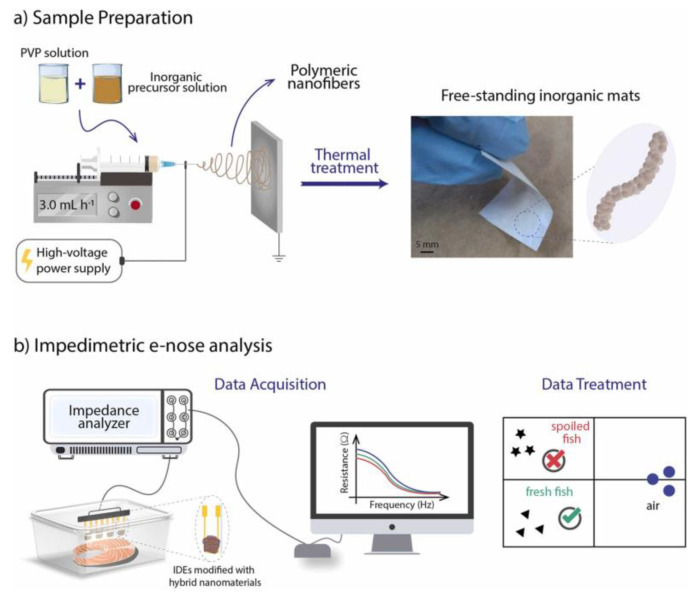
(**a**) Schematic illustration of the electrospinning setup and a digital image depicting the mechanical flexibility of electrospun free-standing inorganic mats. (**b**) Schematic representation of the impedimetric e-nose system using IDEs modified with hybrid nanomaterials for fish spoilage monitoring [170].

Conti et al. prepared anatase-phase TiO_2_ nanostructures via coaxial electrospinning and calcination, mixed with PANI. The TiO_2_/PANI nanocomposite platform exhibited a rapid response to NH_3_ at room temperature (25 °C), with a response time of 55 s to 10 ppm NH_3_ [166]. Liu et al. used a simple and effective single-nozzle electrospinning method to prepare organic semiconductor/insulator core-shell nanofibers from a mixed solution of poly(3,3‴-didodecylquaterthiophene) (PQT-12) and polyethylene oxide (PEO). The chemical sensor with 0.25 ratio PQT-12 nanofibers exhibited a 13% response to NH_3_ at concentrations as low as 50 ppb, with good reversibility and selectivity, and a predicted LOD at the ppb level [167]. Aflaha et al. prepared maltodextrin-coated PVAc nanofibers. At room temperature, the sensor exhibited the highest sensitivity of 0.525 Hz·ppm^−1^, fast response and recovery times of 32 s and 17 s, respectively, and an LOD as low as 1.92 ppm [168]. Rianjanu et al. monitored the spoilage degree of farmed Pacific white shrimp using a QCM gas sensor coated with electrospun PVAc nanofibers. The sensor’s sensitivity to TMA vapor ranged from 0.63 to 1.61 Hz/ppm. The sensor detected spoilage earlier, recording a significant frequency shift (178 Hz) within just 3 h of storage [169]. Andre et al. prepared SiO_2_:In_2_O_3_, SiO_2_:ZnO, and SiO_2_ nanofibers via electrospinning and thermal treatment, followed by modification with PANI and poly(styrenesulfonate). The sensor array performed excellently in monitoring fish spoilage, demonstrating its practical application potential in food freshness monitoring (Figure 11) [170]. Histamine is a potent foodborne toxin, histamine levels in food are often considered freshness markers for monitoring the food quality. Dey et al. fabricated a free-standing hybrid mat based on manganese-cobalt (2-methylimidazole) (Mn-Co(2-MeIm)MOF) and CNF as a non-enzymatic electrochemical sensor to estimate histamine levels for determining the freshness of fish and bananas. The sensor exhibited a wide linear range from 10 to 1500 µM, a low LOD of 89.6 nM, and high sensitivity of 107.3 µA mM^−1^ cm^−2^ [171]. Industrial gas emissions, mining activities, and food spoilage processes generate H_2_S gas, which poses significant health risks. Wang et al. developed a high-performance H_2_S gas sensor by hydrothermal and electrospinning techniques to prepare CuO/Co_3_O_4_ nanoflowers/Co_3_O_4_ nanofibers. The sensor exhibited higher response (194%@25 ppm) and faster response/recovery times (6 s/25 s@25 ppm) at the optimal temperature [172]. Huo et al. grew MoS_2_ nanosheets in situ on the surface of In_2_O_3_ fibers, and the In_2_O_3_@MoS_2_-2 sensor exhibited a high response of 460.61 to 50 ppm H_2_S at room temperature. Furthermore, the sensor was successfully applied to detect oral diseases and food spoilage [173].

### 4.7. Others

Bisphenol A (BPA) is a widespread endocrine disruptor used in the manufacturing of polycarbonate plastics and epoxy resins, which may cause reproductive and neurological damage. Li et al. utilized in situ carbothermal reduction to modify SPCE with ultrasmall gold nanodots supported by CNF (AuNDs@CNFs), significantly enhancing the sensitivity and specificity of BPA detection [174]. Shoukat et al. reported an efficient electrochemical sensor based on CuO-NiO-coated CA/PANI electrospun nanofibers, prepared on a nickel foam electrode (CuO-NiO/CA-PANI@nanofiber), for monitoring of BPA. The nanofibers achieved high sensitivity (0.00172 μA/nM/cm^2^), a low LOD (0.6 nM), a wide linear range (2–100 nM), and high selectivity. The designed electrode was successfully used for the precise monitoring of BPA in bottled water, demonstrating the system’s reliability and practical application potential [175]. Dey et al. synthesized a freestanding nickel–copper pyridine-2,6-dicarboxylic acid (PDA) MOF-anchored carbon nanofiber paper (Ni-Cu(PDA)MOF/CNF). This modification exhibited a linear range of 1 to 150 µmol/L and an LOD of 75 nmol/L, successfully detecting BPA in milk and drinking water samples [176].

Hydrogen peroxide (H_2_O_2_) is widely used in various industries, and determining its concentration is essential for environmental and food safety [177]. Liu et al. synthesized hollow-structured CuO/polyaniline (CuO/PANI) nanocomposite fibers to serve as a novel electrochemical sensor for precise detection of H_2_O_2_. The experimental outcomes revealed that the sensor possesses a wide detection range, a low limit of detection, strong selectivity, and sustained long-term stability [178]. Zhu et al. prepared AgNPs-doped CNF via electrospinning and subsequent calcination. Electrochemical studies showed that the developed sensor had a broad dynamic range of 0.01–50 mM and an LOD of 3 μM [179].

This section presents the latest advancements in electrochemical sensors based on electrospun nanofibers for applications in food safety, with a focus on the detection of heavy metal ions, pesticide residues, food additives, microorganisms and their toxins, freshness indicators, and other contaminants. The primary applications of electrochemical sensors utilizing electrospun nanofibers in food analysis over the past five years are summarized in Table 1. As previously discussed, the small structural dimensions, large specific surface area, and excellent mechanical properties of electrospun nanofibers have significantly enhanced the sensitivity, response speed, and selectivity of electrochemical sensors. The integration of electrospun nanofibers with electrochemical sensing technologies holds great potential for advancing food safety detection methods, providing an efficient and cost-effective solution for safety monitoring across the global food supply chain.

## 5. Conclusions and Outlook

### 5.1. Outlook

Despite the numerous advantages and significant progress of electrospun nanofiber-based electrochemical sensors in food safety analysis, several challenges remain for their practical application. Future research should focus on the following areas:

- **Development of Advanced Nanofiber Materials**: To enhance sensitivity, selectivity, and stability, novel nanofiber materials with improved performance should be developed. The synthesis of high-performance nanomaterials typically requires expertise, and the electrode surface preparation process can be complex and time-consuming. Additionally, the long-term stability of nanomaterials and receptors needs improvement, and there is a limited number of alternative receptors for sensitive and selective detection of specific analytes. Beyond new material development, advanced characterization techniques are crucial for understanding and enhancing the performance of nanofiber-based sensors. Addressing these issues is key to advancing the application of electrochemical sensors.
- **Complexity of Food Matrices**: Food matrices are complex, with food safety hazards often present at low concentrations compared to background substances such as proteins and cells. Freshness detection methods based on volatile spoilage markers are less applicable. The nutritional components and microbial contaminants differ among various types of meat and seafood, resulting in varied levels and compositions of spoilage markers. Moreover, non-specific adsorption of biofouling from sample matrices on the electrode surface reduces the signal-to-noise ratio, severely affecting sensor reliability and accuracy. Research into antifouling materials and antifouling electrode surfaces can mitigate biofouling issues and simplify sample pretreatment, thereby promoting the practical application of electrochemical sensors in real-world testing.
- **On-Site Food Safety Hazard Detection**: Rapid data processing and decision-making are necessary for on-site detection of food safety hazards. The application of nanofiber sensors in portable and field-deployable devices faces challenges, including device miniaturization, power management, and data processing. These sensors need to be integrated into micro platforms to enable real-time monitoring of contaminants. Simplifying detector operations to make systems user-friendly and suitable for portable devices is essential. Combining these sensors with big data platforms and machine learning can enhance data collection and processing capabilities. Machine learning algorithms can improve the detection capabilities of nanofiber sensors, enabling rapid and accurate identification of specific pollutants in environmental samples. Research should focus on developing microfluidic-based systems, energy-efficient power technologies, and data transmission solutions to promote their widespread use in environmental monitoring and toxin detection.

### 5.2. Conclusions

This paper summarizes recent advancements in electrospun nanofiber-based electrochemical sensors for food safety analysis over the past five years. Electrospun nanofiber-based electrochemical sensors represent a novel sensing technology with high sensitivity and rapid response capabilities, occupying a crucial role in food safety detection due to their broad application range and outstanding detection performance. Future research should further explore new nanomaterials, optimize fiber structures, and functionalization strategies to improve sensor performance. Additionally, the development of portable and multifunctional devices will be key to enabling rapid on-site detection, significantly advancing the modernization and intelligence of food safety management. Interdisciplinary collaboration and technological integration will drive progress in this field. With continuous research and innovation, electrospun nanofiber-based electrochemical sensors will play an increasingly significant role in ensuring food safety and enhancing public health levels.

## Figures and Tables

**Table 1:** Analytical performance of various electronic sensors based on electrospun nanofiber for food safety analysis.

Analyte	Material	Linear Range	Limit of Detection	Ref.
Hg^2+^	PA6/CNW:rGO nanofiber	2.5–200 μM	5.2 nM	[75]
Hg^2+^	Aptamer/Au/Pt@CNF	1.0 × 10^−15^–1.0 × 10^−6^ M	3.33 × 10^−16^ M	[77]
Hg^2+^	InZnO nanofiber	10 fM–1 µM	3.13 fM	[78]
Hg^2+^	Ce-ZnO hybrid nanofibers	0.1–100 μM	267 nM	[80]
Hg^2+^	PANFs-CANFs	30–450 nM	9.98 nM	[81]
Cd^2+^	Co/Zn-ZIF nanofibers	100 nM–1 mM	27.27 nM	[88]
Malathion	Nafion/ENCF-67@Au/CQDs	1.0 × 10^−12^–1.0 × 10^−7^ M	3.3 × 10^−13^ M	[99]
Diazinon	Poly(ε-caprolactone)/CHIT nanofibers	3–100 nM	2.888 nM	[104]
Atrazine	SnO_2_ nanofibers	1 zM–1 μM	0.9 zM	[106]
Chlorpyrifos	Fe-PyDA/CNF	1.0–150 μM	15.1 nM	[108]
Methyl parathion	BCL@MOF nanofiber/CHIT	0.1–38 µM	0.067 µM	[110]
Diphenylamine	TiO_2_/Au nanofibers	0.05–60 µM	0.009 µM	[111]
Benomyl	EuVO_4_/CNF	0.125–23.875 µM	0.00612 µM	[114]
Tetracycline	AgZFO/CHIT-CNF	0.2–53.2 μM	1 nM	[117]
Nitrofurazone	TiO_2_/Au-NFs/O-C_3_N_4_	0.008–105 μM	0.001 μM	[118]
Metronidazole	CNF/Fe_2_WO_6_ composite	0.01–1792 μM	0.013 μM	[120]
Acetaminophen	PANI-CoPc-fur-f-MWCNTs	10–200 μM	0.094 μM	[123]
Nitrite	Bi/HCNF	0.1–800 μM	19 nM	[127]
Nitrite	Au WNWs/CNFs-Gr hybrid	1.98 µM–3.77 mM	1.24 µM	[128]
Rutin	MXene-FeWO_4_ nanofibers	4–147 nM	0.42 nM	[129]
Sunset yellow	RuO_2_ nanofibers/PSSA	0.0005–9.0 μM	0.38 nM	[130]
Luteolin	SWCNHs/CNF	0.01–50 μM	3.714 nM	[131]
Vanillin	LCO@CNF hybrid composites	0.01–1670 μM	4.67 nM	[132]
Vanillin	La_2_NiO_4_-CNF	5 nM–3035 µM	6 nM	[133]
Tartrazine	Ni_2_P@f-CNF	0.01–1875 µM	0.011 µM	[134]
Tartrazine	NdVO_4_@F-CNF	0.05–271.6 μM	0.0011 μM	[136]
*Escherichia coli*	Bacteriophages/CNF	10^2^–10^6^ CFU/mL	36 CFU/mL	[141]
*Escherichia coli*	CANF-decorated PBSP	1.5 × 10^2^–10^6^ CFU/mL	30 CFU/mL	[144]
Zearalenone	Bi_2_S_3_@CNF	0.125–375.5 μM 438–1951 μM	0.61 μM	[146]
Aflatoxin M1	ss-HSDNA/AuNPs/ECNF mat	1–100 pg/mL	0.6 pg/mL	[147]
Penicillin	AuNPs/ECNF mat	1–400 ng/mL	0.6 ng/mL	[148]
Ochratoxin A	Ti_3_C_2_T_x_/PVDF nanofiber composites	1 fg/mL–1 ng/mL	2.15 fg/mL	[150]
Aflatoxin B1	Zein/PPy nanofibers immobilized with anti-AFB1 antibodies	0.25–10 ng/mL	0.092 ng/mL	[151]
Trimethylamine	PVAc/CA nanofiber	0.5–2.5 ppm	19 ppb	[157]
Trimethylamine	PAN/PANI fiber membranes	6 ppb–1.1 ppm	6 ppb	[159]
Trimethylamine	ZnWO_4_/ZnO nanofibers	0.5–50 ppm	150 ppb	[162]
Ammonia	Maltodextrin coated PVAc nanofibers	10–250 ppm	1.92 ppm	[168]
Histamine	Mn-Co(2-MeIm)MOF@CNF mat	10–1500 µM	89.6 nM	[171]
H_2_S	CuO/Co_3_O_4_ nanofiber	0.5–100 ppm	500 ppb	[172]
Bisphenol A	AuNDs@CNFs	0.01–50 μM	5 nM	[174]
Bisphenol A	CuO-NiO/CA-PANI@nanofiber	2–100 nM	0.6 nM	[175]
Bisphenol A	Ni-Cu(PDA)MOF/CNF	1–150 µM	75 nM	[176]
Hydrogen peroxide	CuO/PANI nanocomposite fiber	5 μm–9.255 mM	0.110 × 10^−6^ M	[178]
Hydrogen peroxide	AgNPs-doped CNF	0.01–50 mM	3 μM	[179]

## Data Availability

Not applicable.
